# Movement-Specific Reinvestment in Older People Explains Past Falls and Predicts Future Error-Prone Movements

**DOI:** 10.3390/ijerph18105129

**Published:** 2021-05-12

**Authors:** Lisa Musculus, Noel Kinrade, Sylvain Laborde, Melina Gleißert, Miriam Streich, Babett Helen Lobinger

**Affiliations:** 1 Department of Performance Psychology, Institute of Psychology, German Sport University Cologne, 50933 Cologne, Germany; s.laborde@dshs-koeln.de (S.L.); m.gleissert@dshs-koeln.de (M.G.); streich.miriam@gmx.de (M.S.); lobinger@dshs-koeln.de (B.H.L.); 2 SHAPE Research Group, School of Science and Technology, Nottingham Trent University, Nottingham NG18 5BH, UK; Noel.Kinrade@ntu.ac.uk; 3 University of Caen, UFR STAPS EA 4260, 14032 Caen, France; 4 Institute of Movement and Sport Gerontology, German Sport University Cologne, 50933 Cologne, Germany

**Keywords:** longitudinal design, fall prevention, ageing, movement, cognition

## Abstract

The tendency to think about or consciously control automated movements (i.e., movement-specific reinvestment) is a crucial factor associated with falling in the elderly. We tested whether elderly people’s movement-specific reinvestment depended on their past falling history and whether it can predict future error-prone movements. In a longitudinal pre-post design, we assessed *n* = 21 elderly people’s (*M*_age_ = 84.38 years, *SD* = 5.68) falling history, movement-specific reinvestment (i.e., Movement-Specific Reinvestment Scale), and physical functioning (i.e., Short-Physical-Performance Battery). Following a baseline assessment, participants reported their movement behavior in a daily diary for 2 months, after which we assessed their movement-specific reinvestment and physical functioning again (longitudinal, pre-post design). Results revealed, first, that participants’ movement self-consciousness score was fairly stable, while their conscious-motor-processing score was less stable. Second, conscious motor processing was higher in participants who had fallen as opposed to those who had not fallen in the past. Third, conscious motor processing predicted error-prone future movement behavior reported in the daily diary. For identifying individuals who are more prone to fall, caregivers, rehabilitation staff, or doctors could apply the Movement-Specific Reinvestment Scale to screen elderly people’s psychomotor behavior. Based on conscious motor processing, monitoring cognitions could be tailored in theory-based, individual interventions involving both cognitive and motor training.

## 1. Introduction

Preventing falls and encouraging mobility are two of the most important factors for psychosocial well-being in old age [1,2]. From the age of 60 years, however, 30–60% of people fall, resulting in a rate of 0.7–1.6 falls per year for a person in that age group [3]. In an aging society, this has extensive consequences not only for the individual but also on a socioeconomic level [4,5]. Falls of older people lead to high expenses for government and health insurance companies, and an increased workload for nurses, relatives, friends, or neighbors. It is estimated that in-patient falls alone cost the National Health Service in the U.K. £630 million, with falls among older groups accounting for approximately 77% of total reported falls and about 87% of total costs [6]. On an individual level, falls unfortunately often lead to severe injuries that hinder older people’s ability to keep up their everyday life routines, mobility, or independent living, all key components of high quality of life [3,7,8,9,10]. This is why the prevention of falls is highly relevant for aging individuals as well as for aging societies. Detecting deficits in motor control and related error-prone movements might enable the elderly to better cope with age-related changes by developing individual movement-related risk management [11]. For effective risk management in older people, monitoring error-prone movement behavior is crucial, because it still has the potential to be positively influenced through interventions. Error-prone movement behavior includes stumbling, falling, and falling with injuries, which can be assessed through observation or self-reporting [11]. Therefore, we investigated the role of psychomotor factors in explaining past falling history and in preventing error-prone movement behavior. To do so, we applied a longitudinal design with a pre- and post-test.

Falls in older people are caused by multiple physiological and psychological factors [12,13,14]. In this study, we focus on the psychological aspects of movement execution and monitoring, which are associated with past and future motor behavior and resulting falls [10]. Social–cognitive factors such as older people’s fear of falling or their movement self-efficacy are positively related to physical functioning and physical parameters associated with falls, such as functional balance and mobility [14,15,16,17,18,19]. In particular, high fear of falling and low movement self-efficacy negatively impact spatial and temporal parameters, leading to an increased chance of actually suffering a fall [14,15,16,17,18,19]. As a result, older people engage in fewer activities and may experience a lower quality of life [10,15,16,17,18,19,20].

While the influence of such social–cognitive psychological factors on error-prone movements has been empirically established, the influence of psychomotor factors has rarely been considered (for exceptions see [21,22,23,24]). However, considering psychological factors closely related to movement execution seems a promising approach: Studying people’s thoughts or expectations regarding their own movements might not only explain error-prone movement behavior but could also be a way to explore the underlying mechanism of this specific motor-function failure. As falls often happen during highly automatized movements such as walking or running, it might be that movement failure is due to cognitive control while moving [20,22,24,25]. Instead of performing an automatized movement, people seem to consciously control movement execution, which results in performance decrements. They invest cognitive resources in the online control of movements that should normally be highly automatized [21,22,26,27]. Researchers have consistently shown an association between fear of falling and the adoption of stiffening strategies, whereby individuals attempt to control their movements by reducing the range of motion by co-contracting the major muscle groups involved in walking, resulting in lower amplitude and higher frequency of postural sway [28,29]. To clarify how the interrelation of motor and cognitive factors contributes to error-prone movement, their influences should be investigated concurrently.

The impact of conscious control on movement execution has been conceptualized through reinvestment. Reinvestment is defined as a manipulation of explicit, conscious, and rule-based knowledge, by working memory, to control the mechanics of movements [30]. In other words, people consciously “reinvest” knowledge and attentional resources in the movement to be executed. The theory of reinvestment states that consciously observing, monitoring, or controlling movements online can hinder the automatic execution of already highly trained movements such as walking [30,31]. Reinvestment related to movement can be assessed with the Movement-Specific Reinvestment Scale [32]. This scale contains two dimensions: The amount of conscious monitoring while executing a movement, represented by the conscious motor processing factor, and the degree of personal concern regarding one’s own movements, reflected by the movement self-consciousness factor [30,32]. Using the Movement-Specific Reinvestment Scale, research has shown the negative impact of reinvestment on movement errors [33].

Empirically, so far, older people’s movement-specific reinvestment tendency has received only limited attention [21,22,23,25,26,34,35,36]. Previous research demonstrated the association between movement-specific reinvestment and cognitive processing, in measures of planning, brain activity, and gaze behavior [25,26,35,37]. Beyond cognition, conscious movement control has also been shown to increase with task difficulty during gait [36]. Furthermore, studies showed that the two factors of movement-specific reinvestment differ in terms of their relationship with age and their explanatory power concerning *past* falls [21,22,23,34]. Regarding age, Laborde and colleagues (2015) [23] showed that older adults had a significantly higher tendency to reinvest movements than younger adults. Examining the movement-specific reinvestment factors revealed that the age differences were driven by the conscious motor-processing factor only, with older adults monitoring their movements less compared to younger adults [23].

Similarly, Young et al. (2016) [38] found that older adults who stop walking when talking, which has been promoted as an efficient clinical tool to predict falls [39], scored significantly higher on the conscious motor-processing factor and not the movement self-consciousness factor, compared to those who keep walking when talking. Regarding past falls, Wong and colleagues (2008) [21] examined older people and compared those who had fallen to those who had not fallen in the past. Participants who had fallen scored higher on both movement-specific reinvestment factors. Further discriminating fallers from non-fallers, the authors found that only the conscious motor-processing factor differentiated the two groups significantly [21]. However, an investigation into attentional focus and gait stability in elderly fallers and non-fallers revealed no relation between movement-specific reinvestment and whether participants had fallen in the past [34]. The different results highlight that the association between movement-specific reinvestment and history of falling in the elderly is inconclusive and warrants further empirical investigation.

In particular, there are several limitations with these studies that should be acknowledged: Wong and colleagues (2008) [21] claimed to “predict” past falling, but it is more appropriate to consider it as “explaining” past falling; from the perspective of considering a time point in the present, the past can only be explained, not predicted. Similarly, de Melker, Worms, and colleagues (2017) [34] compared fallers and non-fallers based on past falling but did not predict *future* error-prone movements. Relatedly, by comparing older to younger adults in a cross-sectional design, as done by Laborde and colleagues (2015) [23], it cannot be estimated whether in older adults reinvestment remains stable across time. Although reinvestment is generally conceptualized as a trait [30], little empirical evidence exists regarding its trait characteristics. From a diagnostic point of view, it is relevant to know about the stability of the construct, especially if the Movement-Specific Reinvestment Scale is to be used as a screening tool to predict older people’s propensity to fall.

More importantly, it is unclear if movement-specific reinvestment has the power not only to explain past falling but also to predict future falls [24]. The present study aims to extend the existing literature [21,22,23,34] by addressing such methodological limitations. A longitudinal, pre-post design was used, with movements monitored via a movement diary for 2 months, rather than relying on self-reports of past falling alone. Furthermore, in addition to examining the association of movement-specific reinvestment to past falling [21,22,34], our study focuses on the relation of movement-specific reinvestment to future movement behavior and its predictive power regarding future error-prone movements. This is a necessary step in assessing the feasibility of the Movement-Specific Reinvestment Scale to be used as a screening tool.

Given the gaps identified in the current literature, we aimed first to examine older people’s tendency to reinvest in the control of movements and the stability of the construct in older people, focusing on the two reinvestment factors, movement self-consciousness and conscious motor processing. Our second aim was to test whether movement-specific reinvestment can explain self-reported past falling, and our third goal was to scrutinize the effect of movement-specific reinvestment on future falling. First, we expected the reinvestment tendency of older people to be fairly stable (range *r* = 0.60 to 0.78; cf. [23]). Second, regarding the history of falling, we predicted that older people who had fallen in the past would show a higher tendency to consciously control movements compared to those who had not fallen in the past (conscious motor-processing factor). Third, we expected people with a higher tendency to consciously control movements (conscious motor processing) to be more likely to show more stumbling, falling, and falling with injuries (error-prone movement behavior) in the near future. To investigate the hypotheses presented, we conducted a longitudinal study in a pre-post design.

## 2. Materials and Methods

### 2.1. Participants

We used GPower to estimate the sample size a priori [40], which revealed a sample of 18 participants (settings: logistic regression, OR = 11.38, power 1-β = 0.80, α = 0.05) based on the most conservative effect size of Wong and colleagues (η^2^ = 0.31 equals an OR = 11.38) [22]. A total of 21 community-dwelling older women and men (18 females, 3 male) were recruited from two homes for older adults where they lived on their own. To be included, participants needed to be at least 65 years old (*M* = 84.38 years old, *SD* = 5.68; age range 75–96 years) and to pass the Mini-Mental State Test. Developed by Folstein et al. (1974) [41], the Mini-Mental State Test is frequently used to test the cognitive functioning of older people. A maximum score of 30 points can be achieved, and scores of 27 or greater indicate no cognitive deficits, which was used as an inclusion criterion. In total, 20 participants completed the post-test.

### 2.2. Measures

#### 2.2.1. History of Falling

The participants’ history of falling was assessed using a questionnaire administered as an interview with the participants. Participants had to be able to indicate when, where, and why they had fallen to be classified as fallers (1 = yes, 0 = no; cf. Wong et al., 2008).

#### 2.2.2. Movement-Specific Reinvestment

The psychomotor construct of reinvestment was assessed using the German version [23] of the Movement-Specific Reinvestment Scale [32]. Including nine items, the scale measures two dimensions of behavior related to movement. Conscious motor processing is measured with four items (e.g., “I try to think about my movements when I carry them out.”). Movement self-consciousness is measured with five items (e.g., “I am concerned about what people think about me when I am moving.”). All items cover statements related to personal motion sequences and were answered on a 6-point Likert scale, ranging from 1 (do not agree at all) to 6 (completely agree). Internal consistency in this sample was α = 0.75 for conscious motor processing and α = 0.80 for movement self-consciousness.

#### 2.2.3. Physical Performance

Physical ability of the participants was assessed via standardized movement exercises based on the Short Physical Performance Battery [42]. The test battery included three types of exercises that reliably measure the physical functions of older people: gait speed, assessed with 4-m walking tests; strength of the lower extremities, assessed with the chair rise test; and static balance, assessed with three standing positions with increasing difficulty, namely parallel, semi-tandem, and full-tandem stand [43].

#### 2.2.4. Movement Diary

To assess movement behavior and fall frequency, a movement diary was used over a period of 2 months, based on a standardized questionnaire [44]. The participants were instructed to fill out the diary every day to capture their daily movement behavior and to avoid recall bias. In the diary, participants were asked to indicate each day if their movements were disrupted in any way. Four categories were provided from which they were able to select: falling including injuries, falling, stumbling, and nothing happened. The category “falling including injuries” was defined as the sudden, unintentional loss of balance and movement to the ground involving subsequent injury. “Falling”, defined as the sudden, unintentional loss of balance and movement to the ground, did not involve any injury. The category of “stumbling” described the threat of swaying involving a loss of stable gait without actually hitting the ground. The last category, “nothing happened”, indicated that motor behavior was stable.

For the statistical analysis examining the relation between movement-specific reinvestment and self-reported movement behavior in the form of the diary, the frequencies of all error-prone movement categories (i.e., stumbling, falling, and falling involving injuries) were computed for 1 month and the total period of 2 months to detect influences of the reinvestment tendency for different time frames. Additionally, the categorical variable error-prone movement behavior was computed, containing information of all three error-prone movement categories (i.e., falling involving injuries, falling, and stumbling) indicating whether or not participants showed error-prone movements (1 = yes, 0 = no).

### 2.3. Procedure

Before the start of the study, written informed consent of the participants was obtained, and the local ethical review board approved the study protocol for human participants. Participants were tested individually, in a room located in their apartment building. After passing the Mini-Mental State Test, the baseline session began with participants completing the Movement-Specific Reinvestment Scale and performing the Short Physical Performance Battery. At the baseline measurement (t1), participants were introduced to the movement diary. To motivate the participants to be compliant, they were given a “Movement Badge” containing their first test results, which they had to bring to the post-test to have it completed, and the benefits of documenting their movement behavior for the next 2 months were highlighted (e.g., better overview of their movement performance on a day-to-day basis; basis for individual diagnostics and interventions for preventing falls in the future). Further, participants were called by the investigator after 1 month to check if the procedures were clear, clarify any questions that had arisen, and to motivate them to continue completing the diary entries. After 2 months, participants were tested again in a post-test (t2), performing the Movement-Specific Reinvestment Scale and the Short Physical Performance Battery.

### 2.4. Data Analysis

For the first hypothesis, the stability of the two reinvestment factors from t1 to t2 was estimated using Pearson correlation coefficients as an indicator of test–retest reliability. The second hypothesis, whether movement-specific reinvestment (i.e., conscious motor processing, movement self-consciousness) explained the history of falling was tested via independent *t*-Tests comparing people who had fallen to people who had not fallen in the past. For the third hypothesis, the relation of self-consciousness at t1 and conscious motor processing at t1 to the frequency of stumbling and error-prone movements reported in the movement diary was tested using Kendall’s tau. Finally, the predictive power of the reinvestment factors for error-prone movements (yes or no) was checked using logistic regression analyses. The enter procedure was applied and *R*^2^ after Nagelkerke was estimated. After controlling for age and gender differences at step 1, both reinvestment factors were added at step 2. Before conducting the statistical analyses, the assumptions were checked in the data set. Assumptions were met for all predictors.

## 3. Results

### 3.1. Movement-Specific Reinvestment in Older People

Descriptive statistics for both reinvestment factors and the total movement-specific reinvestment score of older people participants are displayed in Table 1. The stability of the reinvestment construct was fairly high for the total movement-specific reinvestment score (*r*_tt_ = 0.62, *p* < 0.001) and the movement self-consciousness factor (*r*_tt_ = 0.71, *p* < 0.001). For the conscious motor-processing factor, the correlation of 0.51, *p* < 0.001, revealed a lower stability score.

### 3.2. Conscious Motor Processing Explaining History of Falling

A review of the participants’ falling history showed that 19% (*n* = 4 females) had not fallen and 81% (*n* = 3 males, *n* = 14 females) had fallen in the past. The descriptive statistics for both groups, fallers and non-fallers, are presented in Table 2. Controlling for gender and age differences revealed that fallers and non-fallers did not differ significantly regarding the distribution of male and female participants, χ^2^(1) = 1.38, *p* = 0.240, nor regarding age, *t*(19) = 1.4, *p* = 0.335. Furthermore, fallers and non-fallers did not differ in any of the objective movement parameters assessed by the Short Physical Performance Battery, all Mann–Whitney *U* = 18.00 to 36.50, all *p* > 0.05.

### 3.3. Conscious Motor Processing Related to and Predicting Error-Prone Future Movements

The movement diary revealed that after 1 month *n* = 3 people had fallen and injured themselves, 1 person had fallen and *n* = 10 people had stumbled several times (*M* = 4.30, *SE* = 2.27). After 2 months, 1 more person had fallen (*n* = 2) and *n* = 12 people had stumbled several times (*M* = 5.33., *SE* = 2.96).

Regarding the relation of movement-specific reinvestment and future error-prone movements, the results supported Hypothesis 3: Correlational analyses revealed that the conscious motor-processing factor was significantly related to future error-prone movements after 1 month, τ = 0.417, *p* < 0.05, and after 2 months, τ = 0.312, *p* < 0.05, while the relation with movement self-consciousness factor was not significant (1 month: τ = 0.10, *p* > 0.05; 2 months: τ = 0.13, *p* > 0.05). Further, the predictive power of the reinvestment factors was checked, controlling for age and gender at step 1 of the logistic regression analyses. First, results showed a nonsignificant model fit to the data when the control variables were included only to predict error-prone movements after 1 month, χ^2^(2) = 4.71, *p* = 0.095, *R*^2^ = 0.28, and after 2 months, χ^2^(2) = 5.71, *p* = 0.058, *R*^2^ = 0.34. Second, adding movement self-consciousness at t1 did not significantly improve the model fit, which remained nonsignificant after 1 month, χ^2^(3) = 5.11, *p* = 0.163, *R*^2^ = 0.30, and after 2 months, χ^2^(3) = 6.39, *p* = 0.094, *R*^2^ = 0.37. As expected, adding conscious motor control at t1 to the model at step 3 revealed a significant model fit to the data, after 1 month, χ^2^(4) = 11.49, *p* = 0.022, *R*^2^ = 0.58, OR = 9.33, and after 2 months, χ^2^(4) = 10.28, *p* = 0.036, and *R*^2^ = 0.54, OR = 9.33. The final models significantly predicting error-prone movements after one month and after two months are presented in Table 3.

Although a significant model fit to the data emerged, the predictive power of conscious motor processing was only marginally significant (after 1 month, *p* = 0.06; after 2 months, *p* = 0.08). After 1 month 70% of people and after 2 months 75% of people were classified correctly.

## 4. Discussion

Fall prevention is relevant for individuals as well as society. To be effective, interventions need to take into account multiple factors related to error-prone movements of older people [3]. In this longitudinal study, we, therefore, focused on the influence of psychomotor aspects of movement execution of elderly, which have received relatively little attention to date. More specifically, we investigated the role of movement-specific reinvestment in older people in an attempt to further understand the interplay of cognitive-motor control and movement execution resulting in failure of automatic movements.

Regarding the first hypothesis, results revealed that the overall movement-specific reinvestment of older people was fairly stable across the 2-month period. However, the conscious motor-processing factor was less stable, indicating that the factor changed over the 2-month interval, and therefore the prediction of the first hypothesis cannot be fully supported. Whilst no study involving older participants has directly examined the stability of movement-specific reinvestment across a stable condition, indirect comparisons can be made. The study of Laborde and colleagues (2015) [23] indicated that the conscious motor-processing factor was stable, *rtt* = 0.78, even tending to be more stable than the movement self-consciousness factor. These differences in results could be due to the different samples tested (sports students aged 18 to 34 years old) or the duration of the test-retest phases (1 month compared to 2 months).

In contrast, similar patterns of results hinting at sensitivity to changes were found in a study with participants who had experienced a stroke [45] and in one of the only studies involving older participants [23]. Partial support of our findings comes from Young et al. (2016) [38], who found anxiety-related increases in both factors of the MSRS in a threat condition during a walking task. It could be hypothesized that in the present study, as the frequency of falls increased across the 2 months, fear of falling and associated state anxiety would also increase, thus supporting the evidence from several researchers that have identified a causal relationship between anxiety and reinvestment [20,21,22,37,38,46].

Our second hypothesis was supported: Conscious motor control differed between fallers and non-fallers, with fallers having a higher reinvestment tendency, indicating that this factor of movement-specific reinvestment can explain past falling of older people. In contrast, movement self-consciousness and the physical functioning of the older people assessed by the Short Physical Performance Battery did not differ between fallers and non-fallers. These results are in line with the findings of Wong and colleagues (2008) [21] showing that past falling status was predicted by the conscious motor-processing factor. Thereby, our results add to the evidence in favor of the relation between movement-specific reinvestment and (past) falling [21] and contrast findings indicating no relation [34,47]. Importantly, while former studies investigated the correlation to past falling [21,34], the present study expands those results to predicting future-movement behavior, which is important for the prevention of falls. These findings also support those by Young et al. (2016) [38], who found that older adults who stopped walking when talking, which has been shown to be associated with future fall risk [39], scored significantly higher on the conscious motor-processing factor and not the movement self-consciousness factor. The group of older adults who stopped walking when talking also showed increases in anxiety and reduced visual-search recall, indicating that older adults more prone to falls are prioritizing conscious motor processing at the expense of external awareness. Consequently, reductions in visual previewing of intended paths have also been shown to be associated with increased stepping errors [20,48] and reduced stepping accuracy [26]. Whilst the Short Physical Battery did not reveal discriminant differences between high and low MSRS scores, future research should look to further examine differences in perceptual capabilities such as visual acuity and visual search strategies. This is also supported by a recent study underlining that gaze behavior was associated with anxiety [26] and might therefore have an indirect influence on error-prone movements [20,48].

The study was longitudinal, and movement behavior was monitored via a movement diary for 2 months after the baseline test. Therefore, falls did not have to be reported retrospectively, which reduced recall bias, and future error-prone movements could be predicted. Results confirmed the third hypothesis, showing that conscious motor processing was also positively related to and predicted error-prone movements, but movement self-consciousness was not. Whilst the Movement-Specific Reinvestment Scale is often viewed as a unidimensional construct [37], the present study adds support to the notion that each factor differs in terms of its role in the allocation of attention during motor performance [38]. It was shown that in older people the two movement-specific reinvestment factors differed in stability. The less stable nature of the conscious motor-processing factor seems to suggest more state-like characteristics in comparison to the stable trait-like factor of movement self-consciousness. Therefore, the results of the present study indicate that future studies should carefully differentiate between the two movement-specific reinvestment factors—movement self-consciousness and conscious motor control—and should examine the state- versus trait-like nature of movement-specific reinvestment for different time frames more closely. Moreover, it should be investigated whether the stability of the construct depends on the population and/or demands of the context. Additionally, future research should aim to understand how the neurophysiological correlates of reinvestment are related to falling, given this may provide additional insights regarding the mechanisms at stake [49].

One limitation of the present study is that it used self-report measures of falling history and online motor behavior reported via a diary. In contrast to standardized questions that are frequently used in studies investigating older people’s likelihood of falling, self-report measures leave the possibility for participants to consciously give incorrect or impartial information. However, the participants were well informed of the importance of answering, and self-report measures are oftentimes used in the investigation of falling history and risks [50]. Further, it should be noted that reflection by the elderly about their movement behavior and potential error-proneness is crucial for their risk management. Therefore, the act of self-report could also be seen as a valuable tool to sensitize older adults for their movements.

Yet another critical point is related to the self-report diary. Although the diary was meant to prevent recall bias, we cannot be sure that the participants always reported the movement behavior daily. Even though we tried to ensure their compliance with the procedure by providing clear instruction and reminder calls, full control was not obtained throughout the 2-month period. In the future, diary studies aiming at monitoring movement behavior should make use of modern experience-sampling techniques, applying movement accelerometers that automatically monitor movement behavior and/or mobile phones that remind people daily to report their behavior and record adherence to the protocol [51].

## 5. Conclusions

Practical implications that can be drawn for the prevention of falls are twofold. First, the results hint at movement-specific reinvestment being a relevant construct explaining the psychological, cognitive control of movement execution and its relation to impaired motor function. Because of the conscious motor-processing factor’s relation to falling history and its power to predict whether error-prone movement patterns will be likely to occur in the near future, it could be used as a screening tool for preventive medical as well as rehabilitation matters. Second, regarding theory-based, individual interventions, this study sheds light on the key role of psychomotor influences. To design interventions, the results of the present study indicate that prevention should focus on the conscious motor-processing factor, meaning the continuous monitoring of movements while executing them. In the study of Laborde and colleagues (2015) [23] involving older people, the conscious motor-processing factor, and not the movement self-consciousness factor, was also related to motor-imagery ability, further supporting this approach. Directly related to motor control, the role of the proprioceptive system playing a crucial role in the positioning of joints could also be considered for prevention [52]. Therefore, to prevent movement failure due to online motor control of automatized movements, these cognitions could be tailored by involving cognitive techniques in combination with mental motor training, for example, motor imagery, for older people [53]. After all, older adults should be encouraged and supported in developing an individual risk-management strategy for preventing falls and maintaining mobility in old age.

To summarize, the present study is the first demonstrating that movement-specific reinvestment, and specifically the conscious processing of movements while they are being executed, is an important factor explaining whether older people fell in the past or will be prone to falls in the future. Future studies should contribute to this line of research and replicate the findings presented in large-scale, potentially community-based research projects involving elderly people. This would provide more empirical support for the use of the MSRS as a diagnostic screening tool as well as a basis for tailoring cognitive and/or mental motor training techniques. The Movement-Specific Reinvestment Scale can consequently serve caregivers, doctors, or physiotherapists as they help older people move safely and efficiently. Therefore, conscious motor processing of older people should be considered when aiming to identify those individuals more prone to future falls and to help structure and design interventions to stabilize older people’s movement.

## Figures and Tables

**Table 1:** Elderly People’s Movement-Specific Reinvestment: Descriptive Statistics.

Factor	Baseline (t1)			Posttest (t2)			t1–t2
	*M* (*SD*)	Min	Max	*M* (*SD*)	Min	Max	*r*_tt_
Movement self-consciousness	3.88 (1.15)	1.60	6.00	3.86 (1.18)	1.60	6.00	0.705
Conscious motor processing	3.50 (1.24)	1.00	6.00	3.44 (1.24)	1.00	6.00	0.510
Total movement-specific reinvestment	3.71 (1.12)	1.33	6.00	3.67 (1.14)	1.33	6.00	0.624

**Table 2:** Movement-Specific Reinvestment Explaining Past History of Falling.

Factor	Past Nonfallers		Past Fallers				
	*M*	*SE*	*M*	*SE*	*t*(19)	*p*	*d*
Movement self-consciousness t1	3.30	0.61	4.01	0.27	1.12	0.139	0.62
Conscious motor processing t1	2.56	0.36	3.72	0.30	1.77	0.047	0.98
Age	83.25	2.78	84.65	1.42	1.4	0.335	0.24

**Table 3:** Results of Logistic Regressions: Movement-Specific Reinvestment Predicting Future Error-Prone Movements After One Month and After Two Months.

Factor	*B*	*SE*	Wald	*df*	*p*
**DV: Error-prone movements after one month**					
Age	−0.08	0.11	0.50	1	0.48
Gender	−21.38	21,211.28	0.00	1	1.00
Movement self-consciousness t1	−1.56	1.17	1.79	1	0.18
Conscious motor processing t1	2.21	1.18	3.53	1	0.06
**DV: Error-prone movements after two months**					
Age	−0.24	0.13	3.19	1	0.07
Gender	−19.66	21,630.80	0.00	1	1.00
Movement self-consciousness t1	−0.64	0.82	0.61	1	0.43
Conscious motor processing t1	1.48	0.86	3.01	1	0.08

## Data Availability

The data are not made publicly available but can be obtained from the authors upon request.
